# Sveinsson Chorioretinal Atrophy: A New Gene Variant

**DOI:** 10.7759/cureus.90588

**Published:** 2025-08-20

**Authors:** Ricardo A Murati Calderon, Sebastián J Vázquez-Folch, Natalio Izquierdo

**Affiliations:** 1 Ophthalmology, University of Puerto Rico, Medical Sciences Campus, San Juan, PRI; 2 School of Medicine, Universidad Central del Caribe, Bayamón, PRI; 3 Surgery, University of Puerto Rico, Medical Sciences Campus, San Juan, PRI

**Keywords:** helicoidal peripapillary chorioretinal degeneration, hippo pathway, novel variant, sveinsson chorioretinal atrophy, tead1

## Abstract

We report the case of a 61-year-old Hispanic female patient with clinical features consistent with Sveinsson chorioretinal atrophy (SCRA), including bilateral peripapillary chorioretinal atrophy and early macular involvement. The patient presented with no significant visual complaints but demonstrated reduced visual acuity in the left eye and characteristic peripheral fundus findings. Multimodal imaging, including spectral-domain optical coherence tomography (OCT), revealed centrifugal extension of atrophy and central macular thinning. Genetic testing identified a heterozygous *TEAD1* variant (c.599C>T; p.Ala200Val), currently classified as a variant of uncertain significance. This variant has not been previously reported in association with SCRA in Hispanic or Caribbean populations. The case highlights the importance of clinical-genetic correlation in the interpretation of rare variants and expands the phenotypic and geographic spectrum of *TEAD1*-associated retinal dystrophies. Broader genetic screening and functional studies are warranted to determine the pathogenic potential of this variant and improve diagnostic accuracy in underrepresented populations.

## Introduction

Sveinsson chorioretinal atrophy (SCRA), also referred to as helicoid peripapillary chorioretinal degeneration, is a rare autosomal dominant retinal dystrophy first described by Kristján Sveinsson in 1939 in Icelandic families [1,2]. Although precise epidemiological data are lacking, SCRA is considered extremely rare and is notably absent from large-scale epidemiologic studies of inherited retinal diseases, suggesting a very low global prevalence. Only a limited number of pedigrees have been identified outside of Iceland, including reports from Serbia and Bosnia [2,3].

Clinically, SCRA is characterized by bilateral, well-demarcated areas of chorioretinal atrophy radiating from the optic nerve head [3]. These lesions primarily affect the retinal pigment epithelium (RPE) and choroid and typically progress in a centrifugal pattern over time to the periphery [4,5]. Patients usually remain asymptomatic in early stages, with peripheral visual field loss preceding central involvement; however, macular extension can lead to progressive decline in central visual acuity [4,5]. 

Mutations in the *TEAD1* gene have been identified as the genetic basis of SCRA [2]. *TEAD1* encodes a transcription factor, a type of protein that binds DNA and helps control when other genes are turned on or off. It functions in the Hippo signaling pathway, a biological system that plays a key role in regulating organ growth, cell proliferation, and retinal development [6]. In the context of SCRA, pathogenic variants in *TEAD1* are believed to disrupt its ability to interact with key transcriptional coactivators such as YAP and TAZ, proteins that act to amplify gene activity, impairing downstream gene activation essential for RPE maintenance and function [7]. This interaction is necessary for proper RPE cell fate and retinal stability, linking *TEAD1* dysfunction to the degenerative process observed in SCRA [6,8,9]. 

We describe a case of a Puerto Rican female patient with a heterozygous *TEAD1* variant (c.599C>T; p.Ala200Val), currently classified as a variant of uncertain significance, who presented with chorioretinal atrophy consistent with SCRA. Furthermore, to our knowledge, this is the first case report with this mutation in the *TEAD1* gene with a phenotype compatible with SCRA in the Caribbean basin. 

## Case presentation

A 61-year-old Hispanic female patient with no significant medical history was referred to our clinic by a retina specialist for a genetic evaluation due to macular atrophy in the left eye (OS). Three weeks before presentation, incidental fundus changes were identified during a routine ocular examination, without preceding visual complaints. The patient denied experiencing ocular complaints such as blurry vision, flashes, photophobia, or nyctalopia. Her ocular history included lattice degeneration in the right eye (OD), and her ocular surgical history was remarkable for argon laser photocoagulation OD and laser-assisted in situ keratomileusis (LASIK) in both eyes (OU). There was no family history of eye disease. Review of systems, toxic habits, and social history was also unremarkable.

Upon comprehensive ophthalmic evaluation by at least one of the authors (NI), the patient’s best corrected visual acuity (BCVA) was 20/25 in OD and 20/50 in OS. Intraocular pressures measured by applanation tonometry were 10 mmHg OD and 9 mmHg OS. Her pupils were equal, round, and reactive to light, with no relative afferent pupillary defect. Extraocular movements were full in all directions of gaze OU and without pain. Color vision and confrontation visual fields were normal OU. Anterior segment examination showed clear corneas with LASIK flaps in place, deep and quiet anterior chambers, and grade 1+ nuclear sclerosis in both lenses. 

A dilated fundus examination revealed bilateral areas of peripheral chorioretinal atrophy radiating from the optic disc centrifugally, consistent with SCRA. In the right eye, there was evidence of superior lattice degeneration with prior laser demarcation, as well as peripheral yellow dots likely representing small drusen and macular drusen. The left eye showed superior peripheral thinning, suggestive of snailtrack degeneration, along with similar peripheral yellow dots and a temporal congenital hypertrophy of the RPE lesion. 

**Figure 1:**
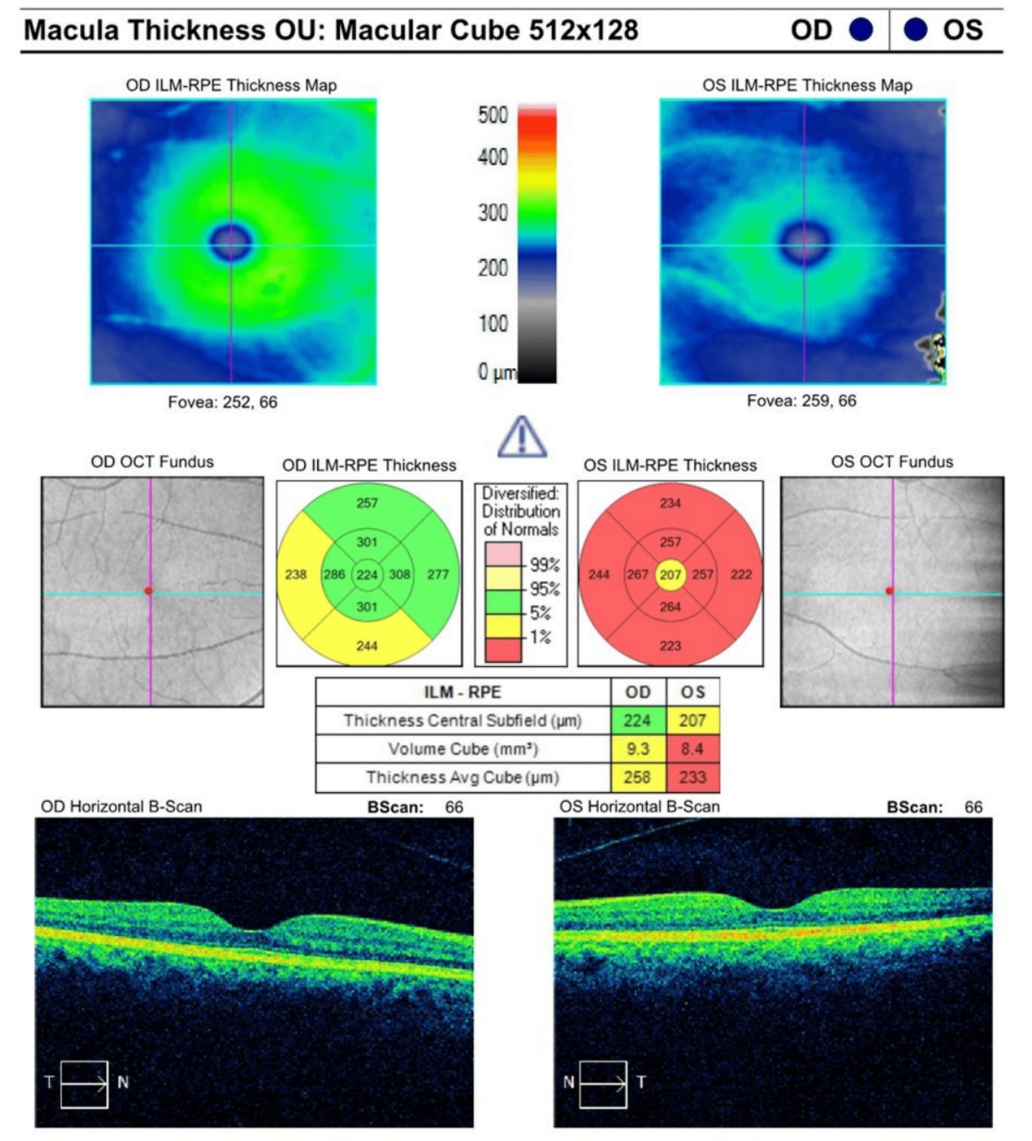
Macular spectral-domain OCT (Cirrus; Carl Zeiss Meditec AG, Dublin, USA) study shows a decreased macular thickness and volume in the left eye (OS) RPE: retinal pigment epithelium, OCT: optical coherence tomography, ILM: inner limiting membrane

Spectral-domain optical coherence tomography (OCT) of the macula (Cirrus; Carl Zeiss Meditec AG, Dublin, USA) (Figure 1) showed a preserved macular contour in the OD with a central subfield thickness (CST) of 224 µm and a cubic volume of 9.3 mm^3^, both within normal limits. In contrast, the OS showed evidence of early macular atrophy, reflected by a reduced CST of 207 µm and decreased cube volume of 8.4 mm^3^. Macular thickness values were within the lowest percentile of normative data, consistent with RPE degeneration and outer retinal thinning. Further, posterior hyaloid separation was present bilaterally, with persistent vitreomacular adhesion noted in the left eye. No cystoid macular edema or subretinal fluid was observed in either eye. 

**Figure 2:**
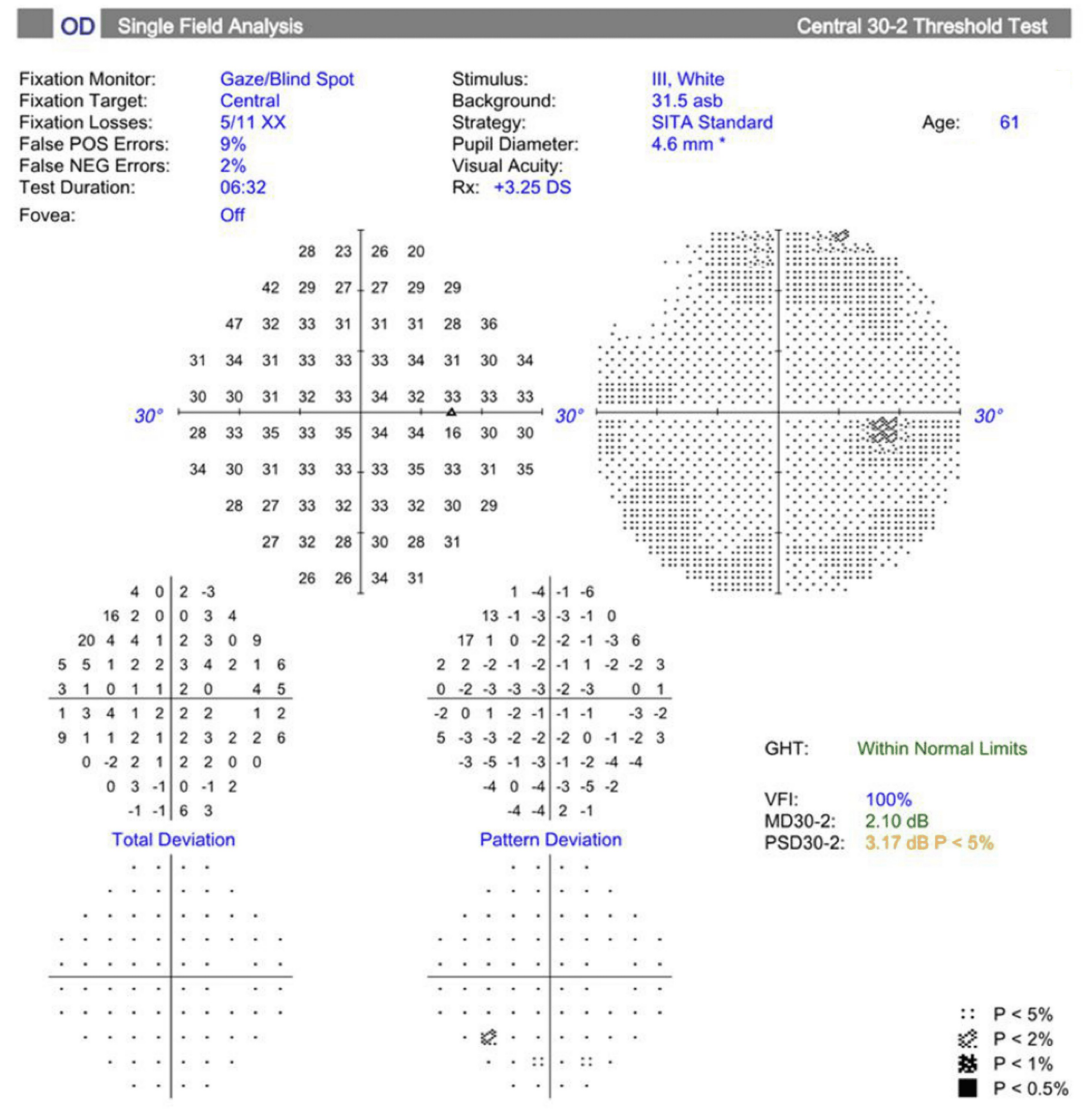
Visual field testing (30-2; Carl Zeiss Meditec AG, Dublin, USA) of the right eye shows an MD of -2.10 dB (p<0.5%) and a PSD of 3.17 dB (p<5%), with a VFI of 100% GHT: glaucoma hemifield test, VFI: visual field index, MD: mean deviation, PSD: pattern standard deviation

**Figure 3:**
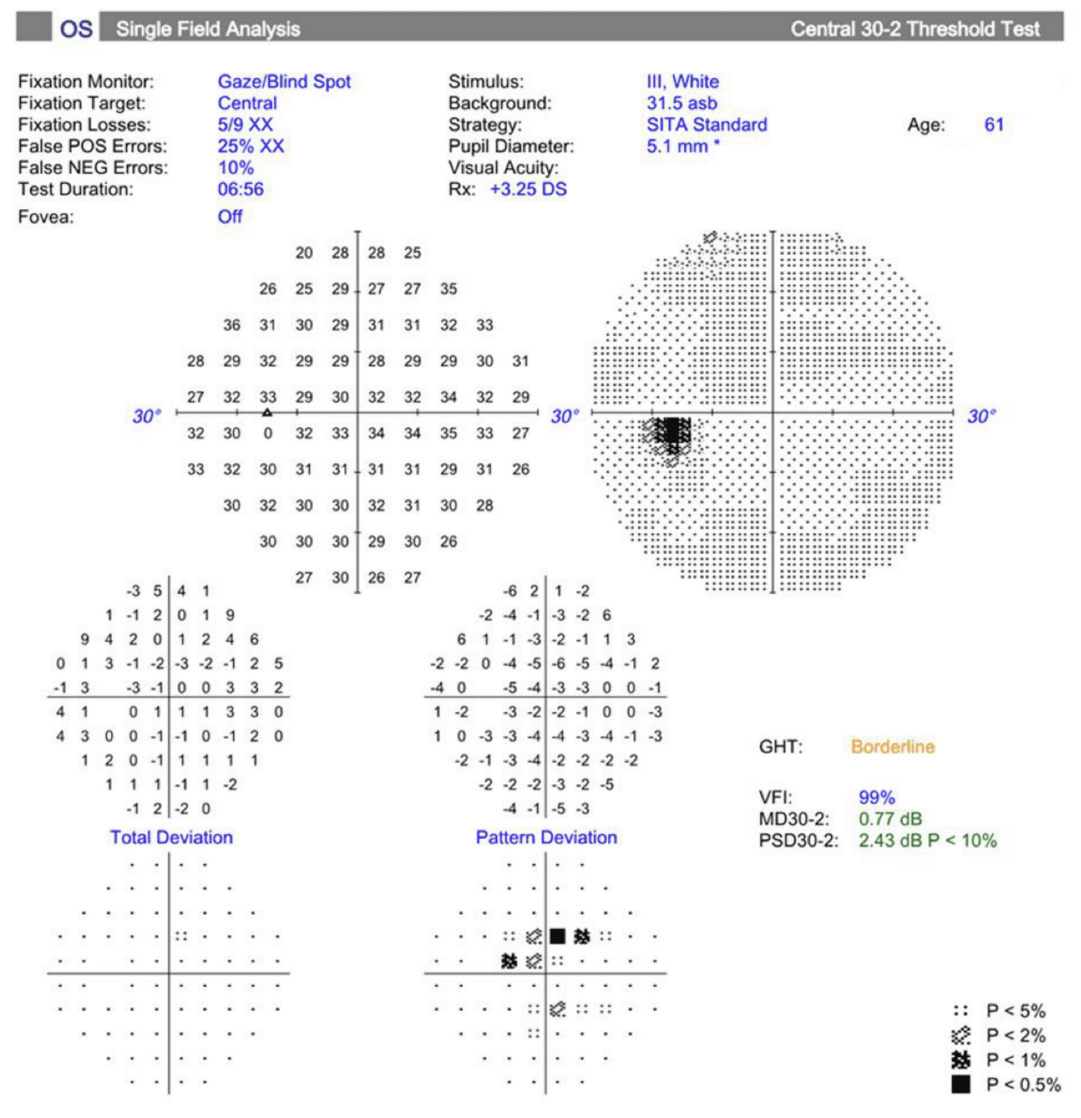
Visual field testing (30-2; Carl Zeiss Meditec AG, Dublin, USA) of the left eye shows an MD of -0.77 dB (p<0.5%) and a PSD of 2.43 dB (p<10%), with a VFI of 99% GHT: glaucoma hemifield test, VFI: visual field index, MD: mean deviation, PSD: pattern standard deviation

As shown in Figures 2-3, upon Humphrey Swedish Interactive Thresholding Algorithm (SITA) Standard visual field analyses (Carl Zeiss Meditec AG, Dublin, USA), the patient had a normal test result in the right eye with no defects, whereas decreased central results were observed in the pattern deviation plots of the left eye. Pattern deviation plots adjust for generalized depression in sensitivity, such as those caused by cataract or media opacity, and highlight areas of localized loss that are more likely to represent true visual field defects. However, both eyes showed unreliable test indices due to elevated fixation losses and high false positive rates. These findings indicate that the patient likely looked away from the fixation target and occasionally responded when no stimulus was presented, which can artificially improve sensitivity values and mask true visual field defects, making the results unreliable. 

Based on the characteristic ophthalmoscopic and multimodal imaging findings, a diagnosis of SCRA was made. Following the diagnosis, the patient was counseled regarding the nature of SCRA, its genetic association with the *TEAD1* gene, and its progressive course. Although no disease-specific treatment exists, a supportive management approach was initiated, focused on residual visual function. The patient was referred for genetic counseling to discuss the implications of the autosomal dominant inheritance pattern and to guide future family screening. Given early macular involvement and reduced macular volume in the left eye, low vision rehabilitation services were recommended to optimize functional vision. Although no evidence supports the use of Age-Related Eye Disease Study 2 (AREDS-2) supplementation in SCRA, given the presence of macular drusen, a trial was initiated to address possible overlapping pathologies. The patient was advised to continue routine follow-up with a retina and genetics specialist for surveillance of structural and functional progression. At the time of the last follow-up, her condition remained stable. 

Genetic testing was conducted using a saliva sample, and a next-generation sequencing (NGS) diagnostic test (Invitae Inherited Retinal Disorders Panel; Invitae Corporation, San Francisco, USA) was performed to evaluate over 330 genes associated with inherited retinal genetic diseases. NGS and deletion and duplication analysis done using the Invitae Panel showed a heterozygous mutation in the *TEAD1* gene. The variant was c.599C>T (p.Ala200Val). This mutation was classified as a variable of uncertain significance.

## Discussion

SCRA is a rare, autosomal dominant retinal dystrophy characterized by helicoid, well-demarcated peripapillary areas of chorioretinal atrophy, classically leading to peripheral vision loss rather than central vision loss in early stages [10]. The disease typically presents in the peripapillary region and progresses centrifugally. However, when the macula becomes involved, central vision loss may occur, as documented by Jonasson et al., who reported a 59-year-old woman with known SCRA and congenital cataracts, who lost central vision in both eyes within a year [4].

Our patient's presentation aligns with the typical features of SCRA, with peripapillary helicoid lesions radiating from the optic disc, but differs in that she demonstrated early macular atrophy in the absence of visual complaints. In the OS, OCT showed thinning of the central macula and reduced cube volume. These findings suggest early macular involvement, which is less commonly recognized in the initial stages of SCRA, and raises the possibility of underrecognized central disease with potential implications for visual prognosis. 

RPE integrity is essential for photoreceptor survival, visual cycle function, and the maintenance of macular thickness [8,9]. Studies have shown that RPE dysfunction is associated with central macular thinning and progressive atrophy, which may contribute to visual loss in SCRA [4]. The functional impact of *TEAD1 *mutations is particularly relevant to our patient, who showed early thinning of the central macula and reduced cube volume. These findings suggest that *TEAD1*-associated RPE dysfunction may directly contribute to central atrophy, supporting previous studies linking RPE pathology to macular structure and function [4,9]. The extension of peripapillary lesions toward the macula may therefore reflect RPE compromise and should not be underestimated as a source of visual decline in SCRA. 

The underlying pathogenesis of SCRA is closely linked to mutations in the *TEAD1* gene, a transcription factor that plays a role in retinal development and homeostasis via the Hippo-YAP/TAZ signaling pathway [3,6]. Beyond its role in RPE maintenance, pathogenic variants in *TEAD1 *may also play a critical role in early ocular morphogenesis, including optic cup formation and closure of the choroid fissure [11]. Disruption of this signaling axis has been implicated in congenital ocular conditions such as SCRA and coloboma, underscoring the essential role of *TEAD1 *in retinal and optic nerve development [11,12]. Ultimately, this disruption can therefore contribute to macular thinning and outer retinal atrophy seen in SCRA [8,9]. 

Our patient carried a heterozygous *TEAD1* variant (c.599C>T; p.Ala200Val), currently classified as a variant of uncertain significance. However, her phenotype, including peripapillary helicoid lesions and early macular atrophy, closely mirrors previously reported SCRA cases with known pathogenic *TEAD1* mutations. In the gnomAD database, this variant is scarce with an allele frequency of 1.80 × 10^-5^ and no reported homozygotes, consistent with its potential relevance in rare monogenic retinal disorders. To our knowledge, this is the first reported case of a Caribbean patient with SCRA and this specific *TEAD1* variant, highlighting a potential expansion in both the genotypic and geographic spectrum of the disease. These findings raise the possibility that while its clinical significance remains uncertain, p.Ala200Val may in fact represent a disease-associated variant, warranting further genetic and functional evaluation. 

Implications for future research include the need for broader genetic screening in underrepresented populations to capture the full spectrum of *TEAD1 *variants and associated phenotypes. Functional assays will be critical to clarify the pathogenic potential of such variants. Additionally, early detection of macular involvement in SCRA could inform prognosis and guide timely referral for visual rehabilitation. 

## Conclusions

Our case represents the first documented report of SCRA in a Caribbean patient carrying a *TEAD1* variant (c.599C>T; p.Ala200Val) with clinical features consistent with the disease. Despite its current classification as a variant of uncertain significance, the patient's phenotype, including bilateral peripapillary chorioretinal atrophy, centrifugal extension toward the macula, and early macular thinning on OCT, closely aligns with previously reported pathogenic *TEAD1* mutations. These findings raise the possibility of an association between this variant and the observed phenotype, particularly given the characteristic clinical features and absence of other contributing systemic factors.

Our findings emphasize the need for careful clinical-genetic correlation in patients with retinal dystrophies, particularly when variants of uncertain significance align with consistent phenotypic expression. Further functional studies and broader genetic screening in underrepresented populations are warranted to clarify the potential association of this variant. Early detection in diverse populations, such as those in the Caribbean Hispanic, is needed. 
